# Evaluation of the patient's perception, reliability and reproducibility, and chairside time with intraoral scanners in adult population—a systematic review

**DOI:** 10.3389/froh.2026.1733387

**Published:** 2026-03-25

**Authors:** Olga Diana Ramos-Morro, Susana Mareque-Ameijeiras, Giovanni Giovannini, Marta Macarena Paz-Cortés, Diego Serrano-Velasco, Juan Manuel Aragoneses, Andrea Martín-Vacas

**Affiliations:** 1 PhD Program in Translational Medicine, San Pablo CEU University, Madrid, Spain; 2 Facultad de Odontología, Universidad Alfonso X El Sabio, Madrid, España; 3 Departamento de Especialidades Clínicas Odontológicas, Facultad de Odontología, Universidad Complutense de Madrid, Madrid, Spain; 4 Department of Dentistry, Faculty of Health Science, Euneiz University, Vitoria-Gasteiz, Spain; 5 Department of Dental Research, Federico Henríquez y Carvajal University, Santo Domingo, Dominican Republic

**Keywords:** dental practices, digital dentistry, digital flow, intraoral scanner, orthodontic

## Abstract

**Introduction:**

Digital impression techniques have gained popularity in dentistry due to their potential advantages in accuracy, efficiency, and patient comfort. This systematic review aims to evaluate and compare the accuracy, chairside time, and patient perception of conventional vs. digital impressions.

**Methods:**

A systematic search was conducted in Medline/PubMed, EBSCO, Web of Science, and Scopus databases. Inclusion criteria comprised clinical or *in vivo* studies comparing conventional and digital impression techniques in terms of accuracy, working time, and/or patient comfort. Reviews, meta-analyses, editorials, and studies involving fully edentulous patients or edentulous spans exceeding two teeth were excluded. Study quality was assessed using the QUADAS−2 tool, and intra-operator agreement was evaluated using the Cohen's Kappa statistic.

**Results:**

From 269 initially identified articles, 10 met the inclusion criteria. All studies assessed accuracy; only two evaluated working time and patient comfort. The included studies, published between 2016 and 2024, were cross-sectional observational in design, with sample sizes ranging from 5 to 50 participants. A variety of intraoral scanners were evaluated, including Cerec, Trios, iTero, and Primescan. The QUADAS-2 tool indicated an overall unclear risk of bias in patient selection and mixed concerns regarding applicability. While findings on accuracy were mixed, most studies concluded that both techniques are clinically acceptable, with conventional impressions performing better in full-arch cases. Digital impressions were consistently reported as faster and more comfortable for patients.

**Limitations:**

The findings of this review should be interpreted with caution due to methodological heterogeneity, small sample sizes, and the inclusion of predominantly young, fully dentate participants.

**Conclusion:**

Intraoral scanners offer advantages in workflow efficiency and patient experience, although conventional impressions remain reliable and widely used. The variability in study designs and outcome measures underscores the need for standardized evaluation protocols in future research.

**Systematic Review Registration:**

https://doi.org/10.17605/OSF.IO/62RCY.

## 1 Introduction

The reproduction of oral structures has long been a fundamental procedure in dentistry. Impression techniques have evolved significantly over time, beginning with the use of beeswax and plaster of Paris in 1856, followed by the introduction of elastomeric materials in 1937 (1), and culminating in the current digital era with intraoral scanners (IOS). Despite continuous improvements in the formulation of conventional impression materials, challenges such as dimensional instability, distortion, and suboptimal organoleptic properties persist (1).

Digital scanning in dentistry was first introduced in 1973, and the first commercially available IOS—CEREC by Dentsply Sirona—was launched in 1987 (2, 3). These devices utilize advanced optics, image sensors, and software algorithms to generate accurate digital models of dental and soft tissue structures (4). Digital dentistry is increasingly becoming standard practice across various dental disciplines, offering benefits such as improved workflow efficiency, enhanced patient comfort, and high precision (4). Moreover, recent technological advancements have enabled IOS to detect caries, monitor tooth wear and soft tissue changes, and incorporate software enhancements that reduce artifacts and noise (5).

Although the literature generally supports the clinical utility of IOS, inconsistencies in study methodologies, measurement protocols, and the variety of IOS brands used make it difficult to draw definitive conclusions about their superiority over conventional techniques (6, 7). Therefore, a systematic review of the current evidence is warranted to determine whether IOS outperform traditional impression methods in terms of accuracy, chairside time, and patient comfort. This systematic review aims to critically evaluate and compare conventional and digital impression techniques to assess whether digital methods can serve as a reliable and efficient alternative in contemporary dental practice.

## 2 Materials and methods

This systematic review was conducted in accordance with the Preferred Reporting Items for Systematic Reviews and Meta-Analyses (PRISMA) 2020 guidelines (8). The research protocol for conducting this systematic review was registered in Open Science Framework (OSF) prior to data extraction and analysis (https://doi.org/10.17605/OSF.IO/62RCY). PRISMA checklist can be consulted in Supplementary Table S1.

### 2.1 Eligibility criteria

The eligibility criteria were defined using the PICO framework:

P (Population): Adolescents and adults aged 18 years and older.
I (Intervention): Intraoral scanners (IOS) used on adult patients.
C (Comparison): Plaster models obtained from adult patients using conventional impressions.

O (Outcomes): Assessment of patient comfort, working time, and accuracy of each technique.

Based on this framework, the inclusion criteria encompassed *in vivo* studies involving adolescent and adult participants (≥18 years) that compared conventional impressions and IOS in terms of comfort, working time, and/or accuracy. Studies published in English or Spanish were considered, with no restrictions on publication date. Exclusion criteria included reviews, systematic reviews, meta-analyses, conference abstracts, editorials, letters, opinion articles, and *in vitro* studies. Studies involving completely edentulous patients or edentulous spans of more than two teeth were excluded, as well as those involving participants with systemic diseases, physical or mental disabilities, limited cooperation, genetic or hereditary disorders, craniofacial abnormalities, or syndromes.

### 2.2 Search strategy

A comprehensive search strategy was developed to identify relevant studies meeting the above criteria. The databases searched included Medline/PubMed, EBSCO, Web of Science, and Scopus. The data search was conducted on November 19, 2024, beginning with Medline (via PubMed), and followed by EBSCO, Web of Science and Scopus.

Search terms were used in English and combined using Boolean operators “AND,” “OR,” and “NOT.” Keywords included: digital impression, intraoral digital impression, intraoral scanning, intraoral scanner, full-arch, alginate, impression, plaster cast, comfort, preference, time, trueness, accuracy, and exclusion terms such as child, teenager, adolescent, implant, extraoral scanners, edentulous, review, and systematic review. Search strategies were adapted to each database's specific requirements. Additionally, the reference lists of included articles were manually screened to identify further eligible studies.

### 2.3 Study selection and quality assessment

The methodological quality of the included studies was evaluated using the Quality Assessment of Diagnostic Accuracy Studies-2 (QUADAS-2) tool (9). Two independent reviewers (O.D.R.-M. and S.M.-A.) screened the titles and abstracts of all retrieved articles based on the predefined eligibility criteria and PRISMA guidelines. Disagreements were resolved through discussion and when an agreement could not be reached, a third researcher (A. M.-V.) acted as an arbitrator. Full-text articles were then assessed for final inclusion, and the QUADAS-2 results were subsequently considered during data synthesis to contextualize the strength and reliability of the available evidence. Before applying the QUADAS-2 tool, both reviewers underwent a calibration process consisting of two pilot assessments on studies not included in the review. Discrepancies were discussed until consensus was reached, and decision rules were documented to ensure consistency. After calibration, the formal assessment was performed independently by both reviewers. To assess inter- and intra-rater reliability, the QUADAS-2 assessment was repeated by both reviewers after a four-month interval. The Cohen's Kappa statistic was used to evaluate agreement levels.

QUADAS-2 tool assesses the risk of bias (RoB) across four domains: patient selection, index test, reference standard, and flow and timing. It also evaluates the applicability of the first three domains to determine the relevance of the study findings to clinical practice. QUADAS-2 was selected because the included studies primarily assessed the performance accuracy of two diagnostic/recording procedures (IOS and conventional impressions), rather than therapeutic interventions, and therefore its domains were considered appropriate to evaluate methodological quality in this context.

### 2.4 Data extraction

A structured data extraction table was developed to collect key information from each study. Extracted data included study characteristics, inclusion and exclusion criteria, materials and methods, statistical analyses, and main findings. To facilitate comparison and quality assessment, the included studies were categorized into three groups based on the primary outcomes evaluated: Patient perception, Scanning and impression time, and Reliability and/or accuracy. This categorization enabled a more systematic evaluation of the evidence across the different dimensions of interest.

### 2.5 Outcome definitions and data synthesis

Accuracy was defined in accordance with the International Organization for Standardization (ISO 5725) as the degree of agreement between a measured value and the true value, encompassing both trueness and precision. Trueness describes how close a measurement is to the reference standard, whereas precision refers to the consistency or repeatability of measurements obtained under the same conditions. Chairside time was defined as the time that it takes to complete each impression procedure. However, articles that measured time were not homogenous and some included tray selection and digitalization as part of the chairside time. Patient comfort was assessed with validated questionnaires. These variations were taking into account when interpreting results.

A meta- analysis could not be done due to the heterogeneity of the outcome measurements. Accuracy was evaluated using a wide range of measurement approaches, such as linear and angular measurements, shell-to-shell deviation analyses, colorimetric evaluations, and qualitative assessments, which were not directly comparable. In addition, chairside time and patient comfort were assessed using different protocols and rating scales. Due to this methodological variability, quantitative pooling of the data was not considered appropriate, and the findings were therefore synthesized qualitatively. A formal assessment of publication bias was not performed because a meta-analysis was not feasible due to methodological heterogeneity.

## 3 Results

### 3.1 Study selection

**Figure 1:**
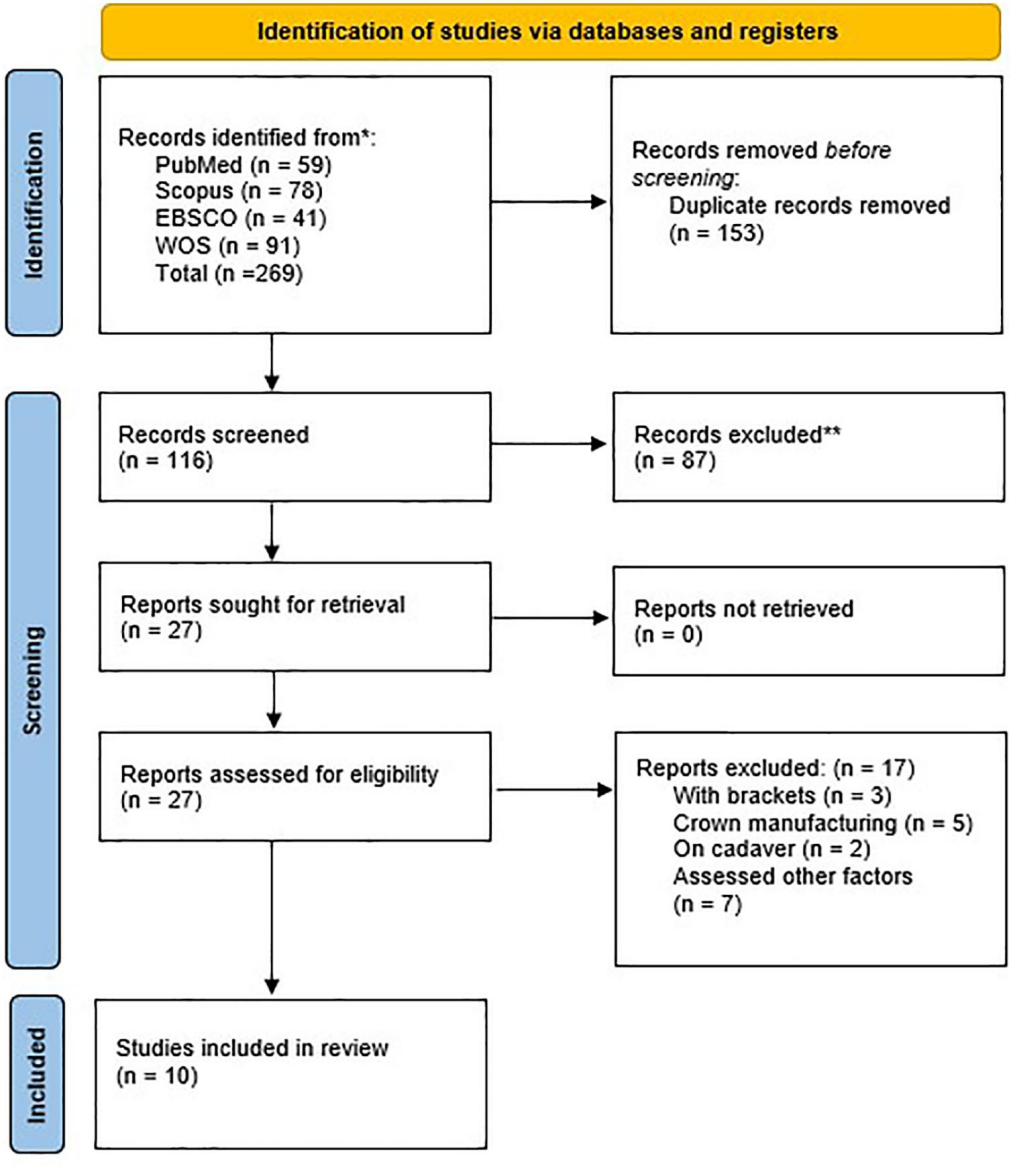
PRISMA diagram of the study selection.

The initial database search yielded a total of 269 articles (Table 1). During the screening phase, 17 articles were excluded (Supplementary Table S2) for the following reasons: three involved participants undergoing orthodontic treatment with fixed appliances; two were conducted on cadaveric maxillae rather than living subjects; five focused on scanning prepared teeth for crown fabrication or scan bodies for implant rehabilitation; and seven addressed topics outside the scope of this review. Following the PRISMA guideline, 10 articles that met the inclusion and exclusion criteria were identified (Figure 1). These studies, published between 2016 and 2024, were ultimately included in the systematic review.

**Table 1:** Search strategy and results of each database during study selection. .

Data base	Search strategy	Results
PubMed	((“digital impression” OR “intraoral digital impression” OR “intraoral scanning” OR “intraoral scanner” OR “intraoral digital scanner”) AND (“full arch”)) AND (alginate OR impression* OR plaster cast)) AND (comfort OR preference OR time OR trueness OR accuracy)) NOT (child OR teenager OR adolescent OR implant* OR “extraoral scanner” OR edentulous) NOT (review (pt) OR “systematic review” (pt)))	59
D&OSS (Ebsco)	(“digital impression” OR “intraoral digital impression” OR “intraoral scanning” OR “intraoral scanner” OR “intraoral digital scanner”) AND (“full arch”) AND (alginate OR impression* OR “plaster cast”) AND (comfort OR preference OR time OR trueness OR accuracy) NOT (child OR teenager OR adolescent OR implant* OR “extraoral scanner” OR edentulous) NOT (review (pt) OR “systematic review” (pt)))	78
WOS	"digital impression” (Topic) or “intraoral digital impression” (Topic) or “intraoral scanning” (Topic) or “intraoral scanner” (Topic) or “intraoral digital scanner” (Topic) and “full arch” (Topic) and alginate (Topic) or impression* (Topic) or “plaster cast” (Topic) and comfort (Topic) or preference (Topic) or time (Topic) or trueness (Topic) or accuracy (Topic) not child (Topic) or teenager (Topic) or adolescent (Topic) or implant* (Topic) or edentulous (Topic) or “extraoral scanner” (Topic) not “review [pt]” (Topic) or “systematic review [pt]” (Topic)	41
Scopus	(TITLE-ABS-KEY (“digital impression”) OR TITLE-ABS-KEY (“intraoral digital impression”) OR TITLE-ABS-KEY (“intraoral scanning”) OR TITLE-ABS-KEY (“intraoral scanner”) OR TITLE-ABS-KEY (“intraoral digital scanner”) AND TITLE-ABS-KEY (“full arch”) AND TITLE-ABS-KEY (alginate) OR TITLE-ABS-KEY (impression*) OR TITLE-ABS-KEY (“plaster cast”) AND TITLE-ABS-KEY (comfort) OR TITLE-ABS-KEY (preference) OR TITLE-ABS-KEY (time) OR TITLE-ABS-KEY (trueness) OR TITLE-ABS-KEY (accuracy) AND TITLE-ABS-KEY (child) OR TITLE-ABS-KEY (teenager) OR TITLE-ABS-KEY (adolescent) OR TITLE-ABS-KEY (“extraoral scanner”) OR TITLE-ABS-KEY (implant*) OR TITLE-ABS-KEY (edentulous) AND NOT TITLE-ABS-KEY (“review”) OR TITLE-ABS-KEY (“systematic review”))	91
Total		269

### 3.2 General characteristics

All included studies were cross-sectional observational designs conducted *in vivo*, with some incorporating an *in vitro* phase. In all cases, the operators did not perform any medical interventions on participants, nor was there any longitudinal follow-up. To minimize variability, most *in vivo* procedures were performed by the same operator on the same day. Notably, Zimmermann et al. (10) was the only study to report randomization during the *in vivo* phase, selecting the arch to be scanned by coin toss. The remaining studies did not specify the order in which impressions were taken, introducing a potential source of bias.

The studies were conducted in Germany (*n* = 5), South Korea (*n* = 2), Italy (*n* = 1), Romania (*n* = 1), and Switzerland (*n* = 1), reflecting a broad international interest in digital impression techniques. However, geographic origin was not considered a limiting factor for methodological quality, as the technologies and protocols used are globally standardized.

Sample sizes ranged from 5 to 50 participants. Zimmermann et al. (10), Schmidt et al. (11), and Ender et al. (12) each included 5 participants; Sfondrini et al. (13) included 14; Lee and Park (14) and Sun et al. (15) included 20; Janosi et al. (16) included 28; Onbasi et al. (17) included 31; and Kuhr et al. (18) and Schmidt (19) each included 50 participants. Several studies (10–12, 15, 18) did not report participant age, referring only to “adults.” In the remaining studies, the mean age was 26.4 years, with a range from 18 to 36 years.

The IOS used for the direct digital impressions were Cerec Omnicam (Sirona, Wals, Austria), Cerec Bluecam (Sirona Dental Systems), Cara TRIOS (Heraeus, Hanau, Germany), Trios 2, Trios 3 Cart wire, Trios 3 Mono, Trios Color, Trios 4 (3Shape, Copenhagen, Denmark), True Definition (3M, St. Paul, USA), iTero (Align Technology, San Jose, CA, USA), Lythos (Ormco Lythos), Carestream CS3600 (Carestream Dental, Atlanta,Ga), Lava COS (3M ESPE, Seefeld, Germany) and Primescan (Dentsply Sirona, Bensheim, Germany). The conventional impressions were taken with different materials irreversible hydrocolloids, medium body polyether, vynylsiloxane, polyvynilsiloxane and addition silicone. These impressions were poured either in dental plaster type 4 or type 2.

### 3.3 Quality assessment

**Figure 2:**
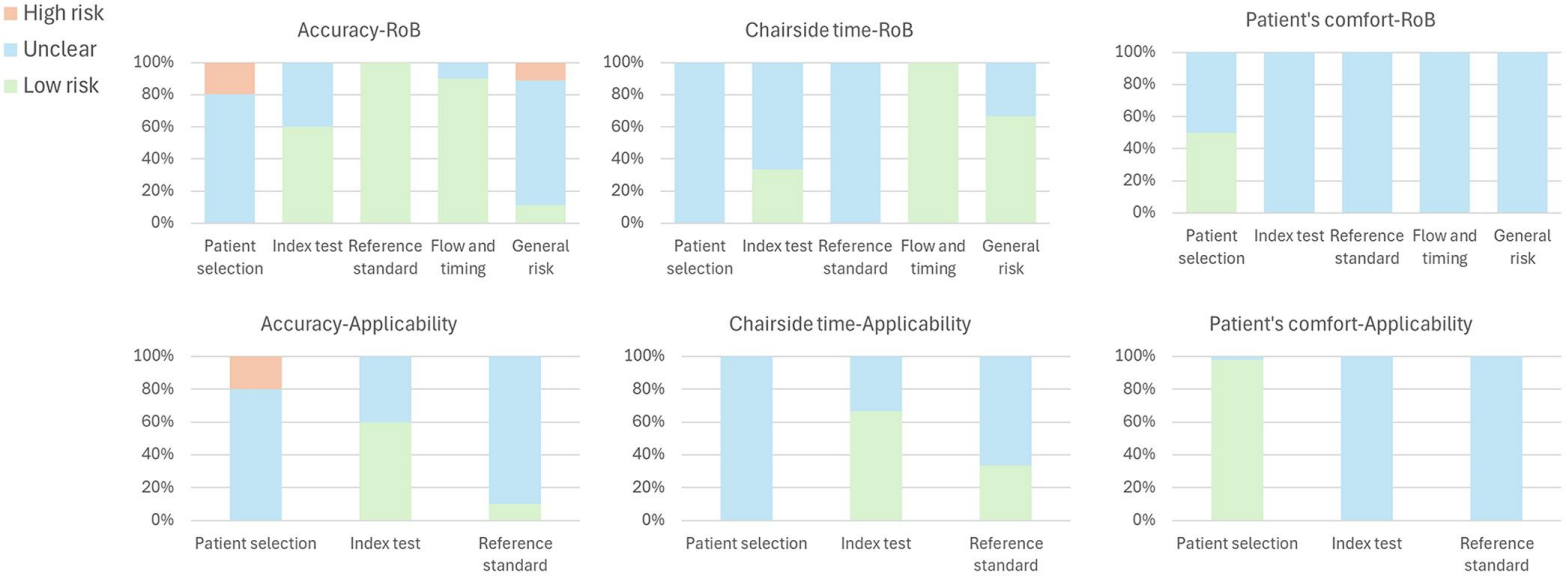
QUADAS-2 results of the included studies.

The final set of selected articles was categorized into three primary outcome domains: accuracy, chairside time, and patient comfort (Table 2). To assess the methodological quality of the included studies, the QUADAS-2 tool was applied (Figure 2). This tool evaluates the risk of bias and concerns regarding applicability across four key domains: patient selection, index test, reference standard, and flow and timing.

**Table 2:** Categorization of the different study areas and the result of the QUADAS-2 tool.

Study	Risk of bias					Applicability		
	Patient selection	Index test	Reference standard	Flow and timing	General risk	Patient selection	Index test	Reference standard
Accuracy								
Zimmermann et al. (10)	High risk	Low risk	Low risk	Low risk	Unclear	High risk	Low risk	Low risk
Schmidt et al. (11)	High risk	Unclear	Low risk	Low risk	Unclear	Unclear	Unclear	Low risk
Ender et al. (12)	High risk	Low risk	Low risk	Low risk	Unclear	High risk	Low risk	Low risk
Sfondrini et al. (13)	Unclear	Unclear	Unclear	Low risk	Unclear	Unclear	Unclear	Unclear
Lee and Park (14)	Unclear	Low risk	Low risk	Low risk	Unclear	Unclear	Low risk	Low risk
Sun et al. (15)	Unclear	Unclear	Unclear	Unclear	Unclear	Unclear	Unclear	Unclear
Janosi et al. (16)	Unclear	Unclear	Low risk	Low risk	Low risk	Unclear	Unclear	Low risk
Onbasi et al. (17)	Unclear	Low risk	Low risk	Unclear	Unclear	Unclear	Low risk	Low risk
Kuhr et al. (18)	Unclear	Low risk	Low risk	Low risk	Unclear	Unclear	Low risk	Low risk
Schmidt et al. (19)	Unclear	Low risk	Low risk	Low risk	Unclear	Unclear	Low risk	Low risk
Chairside time								
Sfondrini et al. (13)	Unclear	Unclear	Low risk	Low risk	Unclear	Unclear	Low risk	Low risk
Janosi et al. (16)	Unclear	Unclear	Low risk	Low risk	Low risk	Unclear	Low risk	Low risk’
Sfondrini et al. (13)	Unclear	Unclear	Unclear	Unclear	Unclear	Unclear	Unclear	Unclear
Janosi et al. (16)	Low risk	Unclear	Unclear	Unclear	Unclear	Low risk	Unclear	Unclear

Most studies (13–19) had an unclear risk of bias in patient selection in terms of accuracy, showing potential limitations in diversity of the sample, as many included young, fully dental arches and without dental restorations or malocclusions. Zimmermann et al. (10), Schmidt et al. (11) and Ender et al. (12) showed a high RoB in this domain due to their small sample size of only five subjects, which significantly limits the reliability of their findings. Regarding the index test, most studies demonstrated a low risk of bias, suggesting well-controlled scanning procedures and the use of at least two IOS. However, Sfondrini et al. (13), Sun et al. (15), Janosi et al. (16) Schmidt et al. (19) had an unclear risk due to the use of only one IOS model. The reference standard was generally low, except for Sfondrini et al. and Sun et al., as the same IOS was used to scan the gypsum models, instead of using a laboratory scan for the digitalization. Flow and timing were considered low risk in most cases, indicating consistent methodology, although Sun et al. (15) and Onbasi et al. (17) showed unclear risk, since the impressions were repeated or done in different dates, suggesting potential inconsistencies in the procedures.

The applicability assessment remained unclear in most studies, mirroring the previous trends. Zimmermann et al. (10) and Ender et al. (12) again presented a high concern in this domain due to their small sample sizes. The index test was generally applicable, with most studies classified as low, but the ones that used just one IOS remained unclear. The reference standard was consistently reliable across studies, with only Sfondrini et al. (13) and Sun et al. (15) showing unclear applicability since the reference model was not digitalized with an IOS.

Among all the studies reviewed, only two assessed chairside time and patient comfort. Sfondrini et al. (13) and Janosi et al. (16) recorded the duration of each procedure. However, as all participants were young, fully dentate individuals without restorations or partial edentulism, the risk of bias and applicability in patient selection remains unclear, as scanning times may vary in more diverse populations. Additionally, both studies relied on a single IOS, further limiting the generalizability of their findings. Both studies were clear on the standard reference and flow and timing, recording the time the same way for each subject and were clear on what was done during that time. These two studies both gave a VAS survey to each patient. However, neither specified whether it was given immediately after each procedure or upon completion of all, potentially introducing bias and uncertainty in applicability. Janosi et al. included a larger sample size which improves results reliability and applicability.

Overall, the methodological quality of the studies was unclear, with some studies showing a RoB in patient selection and the quantity of IOS used. Given this, the results should be interpreted with caution, particularly regarding patient selection and index test.

To assess inter-rater reliability in the evaluation of risk of bias, Cohen's Kappa coefficient was calculated for each domain across two independent reviewers. The agreement was found to be substantial to almost perfect for most items: Patient selection (*κ* = 0.88), Index test (*κ* = 1.00), Reference standard (*κ* = 0.89), and Flow and timing (*κ* = 1.00). The domain General risk showed moderate agreement (*κ* = 0.63). These results indicate a high level of consistency between evaluators, supporting the robustness of the bias assessment process.

### 3.4 Main results

Table 3 shows the main characteristics and outcomes of the studies included.

**Table 3:** Main characteristics of the included studies.

Author, year	Population				Materials & methods					Main results	Main Conclusion
	Sample size	Age		Inclusion and exclusion criteria	IOS	Compared methods	Assessed parameters	Recorded measurements	Statistical analysis		
	*N*	Range	Mean								
Zimmermann et al. (10)	5	NA	NA	Included: full arch	Cerec Omnicam and Lythos	Digitalized dental models taken with alginate impressions	Accuracy	Linear distances and superposition	Kolmogorov–Smirnov One-way repeated measures ANOVA *post hoc* Bonferroni test.	IOS showed significantly lower deviation than alginate impressions (*p* < 0.05)	*In vivo* digital impression is more precise than alginate impressions
Ender et al. (12)	5	NA	NA	Included: full dentition	True Definition, Lava COS, iTero, TRIOS, Trios colour, Cerec bluecam, Cerec Omnicam	Digitalized dental models taken with vynilsiloxanether	Accuracy	Superposition	ANOVA and *post hoc* Bonferroni test	All IOS systems showed deviations <100 µm; conventional impressions showed fewer global deviations	All of the digital impression systems were capable of measuring quadrant impression with clinically satisfying precision. There are differences in precision between different digital impression systems, but while statistically significant, they all fall within a range which allows the successful production of restorations
Schmidt et al. (11)	5	NA	NA	Included: full lower dental arch	TRIOS 3 cart, TRIOS 3 Pod, TRIOS 4 Pod, Primescan	Digitalized dental models taken with polyether	Accuracy	Linear distances between spheres. Superposition of spheres.	Kolmogorov–Smirnov test Levene's test Welch-ANOVA	IOS showed lower deviation for short distances; conventional impressions performed better for long-span distances	Current IOS scanners equipped the with latest software versions demonstrated less deviation for short-span distances but not for long distances
Sfondrini et al. (13)	14	NA	20.4	Included: full dental arch, no temporary teeth, included or supernumerary Excluded: history of mental illness, epilepsy, craniofacial anomalies, dental fear and gag reflex	TRIOS 3 Mono	Digitalized plaster models taken with alginate impressions	Accuracy	Linear distances	Kolmogorov–Smirnov test ANOVA (Analysis of Variance) and Tukey tests Paired *t*-test	No statistically significant differences between IOS and alginate impressions (*p* > 0.05)	Intraoral scanning to acquire data as accurate as alginate impressions
							Time	Conventional: Time 1: included tray selection, mixing, wax bite Time 2: cleaning, disinfection, packaging Time 3: realization of gypsum models. Digital: Times for each sextant and one extra for occlusion		Digital: 5.49 min; Conventional: 22.06 min (*p* = 0.001)	New generation powder-free scanner also reduces both chairside and processing times if compared to alginate impressions
							Patient perception	VAS score		Digital mean score: 9.14; Conventional: 2.57 (*p* < 0.001)	Patients expressed greater comfort and lower gag reflex using the intraoral scanner.
Lee and Park (14)	20	NA	25.5	Excluded: patients with severe crowding, missing teeth and restorative teeth.	iTero and TRIOS	Digitalized plaster models taken with alginate impressions	Patient perception	VAS score	Paired *t*-test Shell-to-shell deviation	No statistically significant differences between IOS and conventional impressions (*p* = 0.206 maxilla; *p* = 0.359 mandible)	Patients expressed greater comfort and lower gag reflex using the intraoral scanner.
Sun et al. (15)	20	NA	NA	Included: full arch from second molar to second molar. Excluded: metal crowns, severe crowding and dentofacial anomalies	TRIOS	Digitalized plaster models taken with alginate impressions	Accuracy	Superposition	Independent *t*-tests. Bland-Altman	*In vivo* scans: 0.04 mm; *ex vivo* scans: 0.02 mm; differences statistically significant but within limits of agreement	*In vivo* and *ex vivo* were comparable but had a slight deviation compared to *ex vivo*
Janosi et al. (16)	28	18–25	21.5	Included: Angle Class I, mild crowding, rotations and spacing. Excluded: systemic health problems, allergies to dental materials, crowns, bridges and orthodontic appliances.	TRIOS	Digitalized dental models taken with alginate	Accuracy	Superposition with best fit algorithm and colour morphometrics	Wilcoxon and Mann–Whitney tests Shapiro–Wilk test GraphPad Prism 9	No significant differences between digital and conventional impressions for full-arch analysis	The intraoral scan can be considered as an alternative to the conventional preliminary impression for performing study model analysis during orthodontic treatment planning.
							Time	Conventional: from the mixing of the material until de digitalization Digital: from the start of the scanner to the import of the file.		Digital: 12 min; Conventional: 75.5 min (*p* = 0.001)	The digital impression has a shorter working time.
							Patient perception	VAS with scale 1–10		Digital mean score: 9.02; Conventional: 6.5 (*p* = 0.001)	The digital impression is more comfortable and accepted by the patients
Onbasi et al. (17)	21	22–32	24	Included: full dental arch with good oral hygiene. Excluded: fixed orthodontic treatment or ongoing dental treatment, caries, periodontitis, severe crowding, dentofacial anomalies or allergy to dental material	Cerec Omnicam TRIOS	Digitalized dental models taken with polyether and addition silicone impressions	Accuracy	Superposition and colour histogram	Wilcoxon signed-rank test	Higher deviations observed in posterior regions; conventional impressions showed lower overall deviation	Digital impression devices can show higher local deviations within the complete arch. More precise results were obtained for the anterior segment compared to the posterior area. Taking into consideration that digital devices should be used with caution in the posterior region.
Kuhr et al. (18)	50	NA	NA	Arch shape that allowed fixation of spheres	Cerec Omnicam True definition Cara TRIOS.	Digitalized plaster models taken with polyether impressions	Time	Conventional: Time 1: included tray selection, mixing, wax bite Time 2: cleaning, disinfection, packaging Time 3: realization of gypsum models. Digital:	Paired sample *t*-test Sign test Bonferroni correction	Conventional impressions showed lower deviation for full-arch measurements (*p* = 0.008)	New generation powder-free scanner also reduces both chairside and processing times if compared to alginate impressions
Schmidt et al. (19)	50	18–36	27	Excluded: subjects with less than 37 mm of mouth opening	TRIOS 3	Digitalized plaster models taken with polyether impressions	Accuracy	Linear measurement and angles	Shapiro–Wilk and Kolmogorov–Smirnov tests (Lilliefors corrected) Levene's test Paired t-tests Wilcoxon test. Effect size (r) Independent t-test	Conventional impressions showed significantly lower deviations in mandibular width	The significant change in the width of the mandible during MMO, which has been partially described in the literature, could not be confirmed in this clinical study.

#### 3.4.1 Accuracy

All included studies evaluated accuracy, though they employed diverse methodologies and measurement criteria. Four studies (12, 13, 18, 19) assessed accuracy by measuring linear distances and/or angular deviations between anatomical landmarks on the models.

Schmidt et al. (11) and Kuhr et al. (18) reported greater deviations in intermolar distances across all methods, while deviations were lower in the intercanine region. In Schmidt et al. (19), conventional impressions outperformed digital methods in most measurements, except for the premolar-to-molar distances on both sides, where digital impressions showed slightly better results. Notably, the highest deviations in digital impressions were observed in intermolar distances. However, no significant differences were found between maximum mouth opening (MMO) and slightly mouth open (SMO) digital models. Comparisons between SMO digital impressions and conventional impressions revealed superior accuracy in the latter for interpremolar, intermolar, and premolar-to-contralateral molar distances. In contrast, Sfondrini et al. (13) found no statistically significant differences between digital and conventional methods.

Kuhr et al. (18) and Schmidt et al. (19) also assessed angular deviations between planes or lines formed by reference points. Kuhr et al. found that only one IOS showed no significant difference compared to conventional impressions (*p* = 0.565), while the remaining IOS yielded similar results among themselves. Schmidt et al. (19) observed that the SMO digital impressions exhibited the highest angular deviations, with conventional impressions performing better in this domain.

All studies, except Sfondrini et al. (13), employed various software tools to superimpose IOS models with digitalized gypsum models, typically using color-coded deviation maps. Studies by Lee and Park (14) and Sun et al. (15) utilized shell-to-shell (S2S) deviation analysis. Lee and Park (14) reported the highest deviations in the molar region, though these were not statistically significant (*p* = 0.206 for the maxilla and 0.359 for the mandible). Sun et al. found statistically significant differences between *in vivo* (0.04 mm) and *ex vivo* (0.02 mm) scans, though both remained within clinically acceptable limits.

Zimmermann et al. (10) and Ender et al. (12) measured surface distances and applied various computational methods to quantify deviations. Zimmermann et al. reported that IOS outperformed alginate impressions in terms of accuracy. Ender et al. found that conventional impressions exhibited fewer overall deviations, although localized discrepancies were noted due to bubbles or tearing artifacts. The IOS used in their study showed deviations primarily at the gingival margins, cusps, or occlusal surfaces, but none exceeded 100 µm. The highest deviations were observed in conventional impressions taken with a Triple Tray, which simultaneously captures upper and lower posterior segments.

Other studies (16–18) employed colorimetric mapping and histograms to visualize deviations. Kuhr et al. (18) concluded that conventional impressions had the lowest overall deviation, with statistically significant differences among most methods (*p* = 0.008), except between two IOS systems. Onbasi et al. (17) found that impressions made with Impregum exhibited the poorest accuracy, both overall and within specific arch segments. In contrast, the TRIOS IOS demonstrated superior performance in full-arch scans. Janosi et al. (16) found no significant differences between upper and lower arches but did observe statistically significant deviations within the same arch, particularly between anterior and posterior teeth in the mandibular arch.

Five studies (11, 12, 17–19) concluded that conventional impressions were more accurate than digital impressions in full-arch cases, particularly in posterior regions and over longer distances. Only Zimmermann et al. (10) reported that IOS were more accurate overall than conventional methods. Schmidt et al. (19) highlighted that IOS performed better over shorter distances, while conventional impressions were more reliable for long-span measurements. Sfondrini et al. (13), Lee and Park (14), Sun et al. (15), and Janosi et al. (16) concluded that both techniques produced clinically acceptable and comparable results for orthodontic analysis and restorative procedures.

#### 3.4.2 Chairside time

Two studies (13, 16) evaluated the time required to complete digital and conventional impression procedures, both reporting significantly shorter chairside times for digital impressions compared to conventional methods. In the study by Sfondrini et al. (13), time was recorded across three distinct phases for conventional impressions: Tray selection, material mixing, and wax bite registration, Cleaning, disinfection, and packaging, and Fabrication of gypsum models. For digital impressions, time was segmented by quadrants scanned and occlusion registration. The mean total time for conventional impressions was 22 min and 6 s, while digital impressions required only 5 min and 49 s, showing a statistically significant difference (*p* = 0.001). When isolating chairside time, the difference was smaller: 7 min and 32 s for conventional impressions vs. 5 min and 49 s for digital. However, the processing time showed a marked contrast, with conventional methods requiring an average of 14 min and 34 s, compared to just 14 s for digital impressions.

In the study by Janosi et al. (16), time for conventional impressions was measured from the preparation of the impression material to the digitalization of the gypsum model. For digital impressions, timing began at the start of the scanning process and ended with the import of the scans into the software. The mean total time was 75.5 min for conventional impressions and 12 min for digital impressions, again demonstrating a statistically significant difference (*p* = 0.001). These findings consistently indicate that digital impressions significantly reduce total procedure time, particularly in the post-processing phase, which may contribute to improved clinical efficiency and patient satisfaction.

#### 3.4.3 Patient perception

Two studies (13, 16) assessed patient comfort using a Visual Analog Scale (VAS) to compare digital and conventional impression techniques. In Sfondrini et al. (13), the VAS questionnaire was administered immediately after each impression procedure, whereas in Janosi et al. (16), the VAS was applied after completion of both procedures. This difference in timing may influence patient-reported comfort scores. In the study by Sfondrini et al. (13), a numerical VAS from 0 to 10 was used, where 0 indicated maximum discomfort and 10 indicated maximum comfort. The questionnaire evaluated five aspects: overall experience, comfort, duration, impression tray dimensions, and gag reflex. In all categories, digital impressions were rated more favorably than conventional impressions. The most significant differences were observed in the comfort and gag reflex domains, both showing highly significant results (*p* = 0.0001). Janosi et al. (16) also employed a VAS, using a scale from 1 to 10, where 1 represented the lowest comfort and 10 the highest. The mean comfort score for digital impressions was 9.02, compared to 6.5 for conventional impressions, with a statistically significant difference (*p* = 0.001), indicating a clear preference for intraoral scanning among participants.

## 4 Discussion

The aim of this systematic review was to analyze the existing literature comparing accuracy, chairside time, and patient comfort between conventional and digital impression techniques in dentistry. A total of 10 articles, published between 2015 and 2023, were included to evaluate whether digital technologies can reliably replace traditional methods in clinical practice.

Although the included studies provide relevant comparative information, the overall available evidence should be interpreted with caution. The QUADAS-2 assessment showed an unclear or high RoB in several domains, particularly in patient selection and, to a lesser extent, in the applicability of the index test. Most studies were conducted on small and well selected samples, frequently involving young, fully dentate subjects. This limited clinical variability, may reduce validity and generalizability of the findings. Furthermore, many studies used one or two IOS and did not randomize the order of the procedures, which may have contributed to influence the accuracy. Consequently, although certain trends can be observed, such as comparable accuracy between digital and conventional impressions and shorter chairside time associated with IOS, the interpretation of these results is conditioned by the methodological limitations.

Accuracy remains a critical factor in selecting an impression technique, as it directly influences diagnostic precision and treatment outcomes. Among the included studies, one favored conventional impressions, one favored IOS, eight reported similar or mixed results, and one was inconclusive. Despite the heterogeneity in measurement methods—ranging from shell-to-shell deviation, color histograms, and point-to-point measurements—a general trend emerged. Several studies (11, 12, 17–19) reported that conventional impressions were more accurate for full-arch scans, particularly in posterior regions and over longer distances. Greater deviations are found over long spans and in posterior regions for IOS, consistent with potential cumulative alignment challenges in full-arch scans. This may be attributed to the image stitching process inherent to IOS, where multiple small scans are merged. Errors in alignment can accumulate, especially in full-arch scans, leading to increased deviations (18).

The type of software and scanning algorithm also plays a significant role in accuracy (11, 12). Updated software versions and optimized scanning protocols can enhance model quality. Additionally, scanner design, such as the size of the handpiece, affects the ability to capture larger areas without introducing artifacts. Performance differences between scanners may relate to device- and software-specific implementations (11, 12, 18, 19), but the included studies did not isolate the effect of handpiece size. Beyond the evidence synthesized, external studies have suggested that ambient lighting may influence IOS trueness although this factor was not directly assessed in the included studies of this review (20). Interestingly, Zimmermann et al. (10) found that IOS outperformed conventional impressions in full-arch accuracy. However, they also noted higher deviations in difficult-to-scan areas, such as steep surfaces, due to the need for multiple image captures and stitching. Conversely, conventional impressions may suffer from local distortions caused by material deformation during removal. Four studies (13–16) concluded that both techniques produced comparable and clinically acceptable results, particularly for orthodontic and restorative applications. Previous literature suggests that deviations between 0.12 mm and 0.30 mm are within acceptable limits for digital impressions (21–24), and the studies included in this review generally fell within this range.

Janosi et al. (16) emphasized that operator technique significantly influences outcomes, regardless of the scanner type. Factors such as saliva, limited mouth opening, patient movement, and tongue position can introduce errors. Koseoglu et al. (20) further highlighted the importance of ambient lighting, noting that controlled lighting conditions and blue scanning light improve accuracy. These findings align with studies that used IOS to scan gypsum models (13, 15), where the absence of patient-related variables resulted in highly accurate *ex vivo* digital models.

Although results are not consistent there is a tendency of IOS giving reliable, equal and sometimes better accuracy than conventional impressions. However, some factors must be considered when interpreting results. Manufacturer instructions on the scanning patterns appear to play an important role as shown in Stefanelli et al. study (25), which concludes that recommended scanning protocols, updated software and improved scanning tips, produce the most accurate models. Nevertheless, important limitations due to the sample size and the subjects accepted into the trial should be acknowledged. All the studies included young subjects without severe dental nor general pathologies, like severe crowding, spacing or edentulism and cleft palates or facial growth deviations. These situations might give different results, and they may favour conventional impressions over IOS. In the systematic review carried out by Srivastava et al. (26) suggests that even though well-maintained bone- supported tissues can be easily scanned, mobile mucosa remains difficult to reproduce digitally, which might make conventional impressions for partial or total edentulism a better option than IOS.

Chairside time was evaluated in two studies (13, 16), both of which reported significantly shorter durations for digital impressions. However, the methodologies differed. Janosi et al. (16) measured the total time from material preparation to model scanning for conventional impressions, while Sfondrini et al. (13) divided the process into three phases: impression taking, post-processing, and gypsum model fabrication. For digital impressions, Janosi et al. measured from scanner activation to scan import, while Sfondrini et al. separated scanning and uploading times. Despite these methodological differences, both studies demonstrated a substantial reduction in total time with IOS [75.5 vs. 12 min (16); 22 vs. 5.8 min (13)]. Notably, the definition of “working time” varied between studies, which may influence direct comparisons. Given that both studies used similar IOS models (3Shape TRIOS 3 and TRIOS 3 Mono) and alginate for conventional impressions, the operator's experience likely played a key role in reducing scanning time. Both studies employed trained operators with moderate experience, but in real-world clinical settings, variability in training and familiarity with IOS may affect efficiency. Therefore, adequate clinician training is essential to fully realize the time-saving potential of digital impressions.

Patient comfort was assessed in the same two studies (13, 16) using VAS questionnaires. Both reported a clear preference for digital impressions. Janosi et al. (16) found mean comfort scores of 9.02 for IOS and 6.5 for conventional impressions, while Sfondrini et al. (13) reported scores of 9.14 and 2.57, respectively. The differences were statistically significant in both cases. The improved comfort associated with IOS may be attributed to shorter procedure times and the absence of bulky trays and impression materials, which are often associated with discomfort and gag reflex. However, Sfondrini et al. (13) noted that some participants experienced discomfort with the scanner tip in posterior regions, although this did not outweigh the overall preference for digital impressions. The timing of patient-reported outcome assessment is a critical methodological factor that can substantially influence the validity of comfort comparisons between impression techniques. Immediate administration of VAS questionnaires, as performed by Sfondrini et al. (13), captures real-time discomfort and gag reflex responses, providing a more accurate reflection of the patient's experience during the procedure. Conversely, delayed assessment, as in Janosi et al. (16), introduces potential recall bias and may attenuate differences between techniques. When evaluations occur after both procedures are completed, short-lived adverse sensations associated with conventional impressions, such as gag reflex, unpleasant taste, or tray pressure, may be underreported, while the overall impression of the second technique may benefit from recency effects or familiarity. This scenario could artificially favor digital impressions, not necessarily because of superior comfort, but due to memory decay and cognitive contrast effects. Furthermore, delayed assessments may also be influenced by social desirability bias, where patients retrospectively rationalize their preference for the more technologically advanced method.

This systematic review presents several limitations that should be acknowledged. First, the heterogeneity in study designs, including differences in measurement protocols, types of IOS, and outcome definitions, limited the ability to perform direct comparisons or meta-analyses. Additionally, the sample populations in the included studies were predominantly composed of young, healthy, fully dentate individuals, which restricts the generalizability of the findings to broader clinical scenarios, such as patients with partial or complete edentulism, malocclusions, or systemic conditions. This inclusion criteria could affect and limit the generalizability of this systematic review. Furthermore, the lack of standardization in the measurement of chairside time and the uncertainty regarding the timing of patient-reported outcome assessments may have introduced bias in the evaluation of patient comfort and procedural efficiency. This systematic review is limited by potential language bias, as only English and Spanish studies were included. Furthermore, publication bias could not be formally assessed because quantitative synthesis was not possible.

Despite these limitations, this review has several strengths. It was conducted in accordance with the PRISMA 2020 guidelines, ensuring methodological rigor and transparency throughout the selection and analysis process. The inclusion of recent studies provides an up-to-date overview of the current evidence on digital vs. conventional impression techniques. Moreover, the use of the QUADAS-2 tool allowed for a structured and critical appraisal of the methodological quality and applicability of the included studies, with high inter-rater agreement supporting the reliability of the assessment process.

The applicability of these findings is primarily limited to young, fully dentate adults without significant restorative work or malocclusion, as these were the predominant characteristics of the included samples. Therefore, results may not be generalizable to elderly patients, individuals with partial edentulism, extensive prosthodontic rehabilitation, craniofacial anomalies, or compromised oral conditions. In such cases, conventional impressions may remain the preferred option due to challenges in scanning mobile mucosa, complex occlusal relationships, and limited oral access. Clinicians can consider IOS as a reliable alternative for orthodontic and restorative procedures in patients with complete dentition and good oral health, given their advantages in workflow efficiency and patient comfort. However, caution is advised when treating patients with complex anatomical or prosthodontic needs until further evidence is available. Future studies should aim to address the current gaps by employing standardized protocols for evaluating accuracy, chairside time, and patient-reported outcomes. Research should also focus on larger and more diverse populations, including patients with complex clinical conditions such as extensive edentulism, craniofacial anomalies, or limited oral access. Additionally, further investigation is needed to assess the influence of operator experience, scanner type, and environmental factors on the performance of IOS. Establishing consensus on clinically acceptable thresholds for accuracy and comfort, as well as developing uniform reporting standards, will be essential to facilitate meaningful comparisons and guide evidence-based clinical decision-making in digital dentistry.

## 5 Conclusion

The findings of this systematic review suggest that IOS can provide clinically acceptable accuracy and improved patient comfort compared to conventional impressions, particularly in young, fully dentate populations. However, these conclusions should be interpreted with caution due to the heterogeneity of study designs, outcome measures, and scanner models, as well as the unclear or high risk of bias in several domains. The evidence base is limited by small sample sizes and the predominance of participants with complete dentition and good oral health, which restricts generalizability to elderly patients, partially edentulous cases, and complex prosthodontic scenarios. While IOS may offer workflow advantages in selected clinical contexts, further research using standardized protocols and more diverse populations is essential before broader recommendations can be made.

## Data Availability

The original contributions presented in the study are included in the article/Supplementary Material, further inquiries can be directed to the corresponding authors.
