# Supplementary material for: Evaluation of the patient's perception, reliability and reproducibility, and chairside time with intraoral scanners in adult population—a systematic review
Source: Front Oral Health. 2026 Mar 25;7:1733387. doi: 10.3389/froh.2026.1733387 (PMC13057287; doi:10.3389/froh.2026.1733387)
Supplement: Supplementary file 1 [file Table1.docx]

**Supplementary Table S1.** PRISMA checklist.

| **Section** | **Topic** | **Item #** | **Checklist item** | **Location where item is reported** |
| --- | --- | --- | --- | --- |
| **TITLE** | **Title** | **1** | Identify the report as a systematic review. | Title page; Abstract |
| **ABSTRACT** | **Abstract** | **2** | See PRISMA 2020 for Abstracts checklist. | Abstract (structured) |
| **INTRODUCTION** | **Rationale** | **3** | Describe the rationale for the review in the context of what is already known. | Introduction: paragraphs 1–3 |
|  | **Objectives** | **4** | Provide an explicit statement of the objective(s) or question(s) being addressed. | Introduction: final paragraph |
| **METHODS** | **Eligibility criteria** | **5** | Specify the inclusion and exclusion criteria, including study design, participants, interventions, comparators, and outcomes. | Methods: Eligibility Criteria |
|  | **Information sources** | **6** | Specify all information sources (e.g., databases, registers) with dates of last search, and indicate whether anyone searched trial registers or contacted authors. | Methods: Search Strategy |
|  | **Search strategy** | **7** | Present full search strategies for all databases, including filters and limits used. | Table 1; Methods: Search Strategy |
|  | **Selection process** | **8** | Specify the methods used to decide study eligibility (selection process), including how many reviewers screened each record and whether they worked independently. | Methods: Study Selection and Quality Assessment |
|  | **Data collection process** | **9** | Specify the methods used to collect data from reports (data collection process). | Methods: Data Extraction |
|  | **Data items** | **10a** | List and define all outcomes for which data were sought, including how and when they were measured. | Methods: Outcome definitions and data synthesis |
|  |  | **10b** | List and define other variables for which data were sought (e.g., participant and intervention characteristics). | Methods: Data Extraction; Results: General Characteristics |
|  | **Study risk of bias assessment** | **11** | Specify the methods used to assess risk of bias in the included studies. | Methods: Study Selection and Quality Assessment |
|  | **Effect measures** | **12** | Specify the methods used to assess certainty (or confidence) in the body of evidence (e.g., GRADE). | Not performed. Stated in Discussion/Limitations |
|  | **Synthesis methods** | **13a** | Describe the processes used to decide which studies were eligible for synthesis. | Methods: Study Selection and Results: Study selection |
|  |  | **13b** | Describe any methods required to prepare the data for presentation or synthesis. | Methods: Outcome definitions and data synthesis |
|  |  | **13c** | Describe methods used to tabulate or visually display results of individual studies and syntheses. | Tables 2–3; Figures 1–2 |
|  |  | **13d** | Describe any methods used to synthesize results and the rationale for the choices. | Methods: Outcome definitions and data synthesis |
|  |  | **13e** | Describe any methods used to explore possible causes of heterogeneity. | Not applicable. Sources of heterogeneity described narratively in Discussion |
|  |  | **13f** | Describe any sensitivity analyses conducted to assess the robustness of the synthesized results. | Not applicable |
|  | **Reporting bias assessment** | **14** | Describe any methods used to assess risk of bias due to missing results (publication bias). | Not performed. Added statement in Methods and Discussion |
|  | **Certainty assessment** | **15** | Describe any methods of additional analysis (e.g., subgroup, meta-regression). | Not applicable |
| **RESULTS** | **Study selection** | **16a** | Describe the results of the search and selection process, ideally using a flow diagram. | Results: Study selection; Figure 1 |
|  |  | **16b** | Cite studies that appeared to meet inclusion criteria but were excluded, and explain why. | Supplementary Table S2 |
|  | **Study characteristics** | **17** | Present characteristics of included studies. | Results: General Characteristics; Table 3 |
|  | **Risk of bias in studies** | **18** | Present risk of bias assessments for each included study. | Results: Quality assessment; Figure 2 and Supplementary Table 2 |
|  | **Results of individual studies** | **19** | For all outcomes, present the results for each study and, if appropriate, effect estimates and precision. | Results: Main results; Table 3 |
|  | **Results of syntheses** | **20a** | For each synthesis, briefly summarize the characteristics and risk of bias among contributing studies. | Results: Quality assessment; Discussion: evidence appraisal |
|  |  | **20b** | Present results of all statistical syntheses conducted. | Not applicable |
|  |  | **20c** | Present results of investigations of heterogeneity. | Not applicable |
|  |  | **20d** | Present results of sensitivity analyses. | Not applicable |
|  | **Reporting biases** | **21** | Present assessments of risk of bias due to missing results (arising from reporting biases) for each synthesis assessed. | Results: Quality assessment |
|  | **Certainty of evidence** | **22** | Present assessments of certainty (or confidence) in the body of evidence for each outcome assessed. | Not applicable |
| **DISCUSSION** | **Discussion** | **23a** | Provide registration information for the review, including register name and registration number, or state that the review was not registered. | Abstract, Methods. |
|  |  | **23b** | Discuss limitations of the evidence included in the review. | Discussion: limitations section |
|  |  | **23c** | Discuss limitations of the review processes used. | Discussion |
|  |  | **23d** | Discuss implications of the results for practice, policy, and future research. | Discussion: last paragraph. |
| **OTHER INFORMATION** | **Registration and protocol** | **24a** | Provide registration information for the review, including register name and registration number, or state that the review was not registered. | OSF registration provided in Methods; protocol registered prior to data extraction. |
|  |  | **24b** | Indicate where the review protocol can be accessed, or state that a protocol was not prepared. |  |
|  |  | **24c** | Describe and explain any amendments to information provided at registration or in the protocol. | Not applicable. |
|  | **Support** | **25** | Describe sources of financial or non-financial support for the review, and the role of the funders or sponsors in the review. | Funding: Not applicable |
|  | **Competing interests** | **26** | Declare any competing interests of review authors. | Conflict of Interest: declared none |
|  | **Availability of data, code and other materials** | **27** | Report which of the following are publicly available and where they can be found: template data collection forms; data extracted from included studies; data used for all analyses; analytic code; any other materials used in the review. | Specified in the manuscript statements. |

**Supplementary Table S2.** Excluded studies during search procedure.

| **Author** | **Manuscript title** | **Reason for exclusion** |
| --- | --- | --- |
| **K.E. Ahmed et al. (2018)** | Performance and perception of dental students using three intraoral CAD/CAM scanners for full-arch scanning | Performed on a mannequin |
| **Nagy et al. (2020)** | Comparing the trueness of seven intraoral scanners and a physical impression on dentate human maxilla by a novel method | Performed on a cadaver maxilla |
| **Vág et al. (2020)** | Marginal and internal fit of full ceramic crowns milled using CADCAM systems on  cadaver full arch scans | Performed on a cadaver |
| **Ahrberg et al. (2015)** | Evaluation of fit and efficiency of CAD/CAM fabricated all-ceramic restorations based on direct and indirect digitalization: a double-blinded, randomized clinical trial | Measurements were con CADCAM produced prosthetics |
| **Christopoulou et al. (2022)** | Patient-reported experiences and preferences with intraoral scanners: a systematic review | Systematic Review |
| **Haddadi et al. (2018)** | Patient-reported experiences and preferences with intraoral scanners: a systematic review | Study performed for CAD CAM prosthetic production |
| **Bilir et al. (2020)** | Comparison of Digital and Conventional Impression Methods by Preclinical Students: Efficiency and Future Expectations | Performed on a single prepared tooth |
| **De Angelis et al. (2017)** | Monolithic zirconia and digital impression: case report | Performed for CAD CAM prosthetic production |
| **Keul et al. (2019)** | Accuracy of full-arch digital impressions: an in vitro and in vivo comparison | Partial mouth on one patient and casting with resin |
| **Alfallaj et al. (2022)** | Procedure Time and Students’ Perception Comparing Full Arch Digital Scans with Conventional Impressions: A Cross-Over Randomized Experimental Trial | Performed on a prepared tooth of a typodont |
| **Schmalzl et al. (2023)** | The effect of generation change on the accuracy of full arch digital impressions | Studies generation differences on the usages of technology |
| **Kwon et al. (2021)** | Full-arch accuracy of five intraoral scanners: In vivo analysis of trueness and precision | No comparison with conventional models |
| **Morsy et al. (2022)** | In vivo precision of digital static interocclusal registration for full arch and quadrant arch scans: a randomized controlled clinical trial | Focuses on bite registration accuracy and not arch accuracy |
| **Bhatia et al. (2024)** | Evaluation of the accuracy of full-arch impressions between three different intraoral scanners and conventional impressions: A prospective in vivo study | Used underaged subjects |
| **Bock et al. (2023)** | What to Prefer in Patients with Multibracket Appliances? Digital vs. Conventional Full-Arch Impressions—A Reference Aid-Based In Vivo Study | Performed on subjects with fixed orthodontic appliances in place |
| **Schlenz et al. (2020)** | Digital versus Conventional Impression Taking Focusing on Interdental Areas: A Clinical Trial | Focused on interdental display instead of impression accuracy of full dental arch |
| **Pellitteri et al. (2022)** | Comparative analysis of intraoral scanners accuracy using 3D software: an in vivo study | Included underaged subjects |
